# Soluble TREM2 is a potential biomarker for the severity of primary angiitis of the CNS

**DOI:** 10.3389/fimmu.2022.963373

**Published:** 2022-12-27

**Authors:** Tianshu Guo, Jia Ma, Jiali Sun, Wangshu Xu, Hengri Cong, Yuzhen Wei, Yuetao Ma, Qiaoxi Dong, Yunting Kou, Linlin Yin, Xinghu Zhang, Haoxiao Chang, Huabing Wang

**Affiliations:** ^1^ Department of Neurology, Neuroinfection and Neuroimmunology Center, Beijing Tiantan Hospital, Capital Medical University, Beijing, China; ^2^ Department of Neurology, Beijing Shunyi Hospital, Beijing, China; ^3^ China National Clinical Research Center for Neurological Diseases, Beijing Tiantan Hospital, Capital Medical University, Beijing, China; ^4^ Institute for Physical Activity and Nutrition, School of Exercise and Nutrition Sciences, Deakin University, Melbourne, VI, Australia; ^5^ Department of Biomedicine, Beijing City University, Beijing, China

**Keywords:** primary angiitis of the central nervous system, sTREM2, cerebrospinal fluid, serum, neuroinflamation, microglia

## Abstract

**Background:**

Primary angiitis of the central nervous system (PACNS) is a severe inflammatory disease, and soluble triggering receptor expressed on myeloid cells 2 (sTREM2) has been reported to be associated with inflammation of the CNS. However, the role of sTREM2 in PACNS remains unknown.

**Methods:**

We obtained serum and cerebrospinal fluid (CSF) samples from 18 patients diagnosed with PACNS, as well as 14 patients diagnosed with other neurological disorders with no evidence of inflammation. sTREM2 concentrations in the samples were detected by enzyme-linked immunosorbent assay. And routine CSF measurements of PACNS patients were analysed, including number of White Blood Cells (WBC), protein, Immunoglobulin G (IgG) index and CSF/serum quotients. Levels of inflammatory cytokines, including tumor necrosis factor-α, interleukin (IL)-6, IL-8, IL-1β, and complement C4, also were tested. The modified Rankin scale (mRS), National Institutes of Health Stroke Scale (NIHSS), and activities of daily living (ADL) scores were obtained as indicators of disease severity. In PACNS patients, cerebral lesion volume was evaluated by magnetic resonance imaging.

**Results:**

sTREM2 levels in serum and CSF were significantly elevated in PACNS patients and significantly associated with the mRS, NIHSS and ADL scores as well as inflammatory cytokine levels. Additionally, positive correlations were observed between the cerebral lesion volume and the sTREM2 levels in both blood and CSF. Higher sTREM2 levels in either the blood or CSF seemed to predict a good prognosis in PACNS patients.

**Conclusion:**

Our results indicate an association between serum and CSF sTREM2 levels and the severity of neurological damage. Thus, sTREM2 represents a potential biomarker for monitoring disease and potentially predicting the prognosis of PACNS patients.

## Introduction

The characteristic histologic feature of primary angiitis of the central nervous system (PACNS) is inflammation marked by mononuclear cells mainly at the small-to-medium vessels of the CNS parenchyma and leptomeninges (1, 2). PACNS is a severe and rare neuroinflammatory disease with an incidence of 2.4 cases per one million per year (3). The main clinical symptoms are headache, focal neurological deficits, epileptic seizures, cognitive dysfunction and psychiatric symptoms, and additional rare manifestations include myelopathy, optic neuritis and ataxia (4, 5). At present, biopsy is the gold standard for PACNS diagnosis (5). Other than biopsy, imaging is an important method for diagnosing PACNS, and the most sensitive imaging technique for PACNS is magnetic resonance imaging (MRI) (6). Patients with PACNS may present with multiple infarctions involving cortex and subcortex gadolinium-enhanced lesions on MRI (6–8), and relapsing-remitting clinical course is common. Currently, the main treatment is immunosuppressive therapy with agents such as glucocorticoids and cyclophosphamide, but even with such therapy, nearly 50% of patients experience relapse (5, 9, 10). To date, the pathogenesis of PACNS has remained unclear due to the low prevalence and poor prognosis of the condition (11).

Triggering receptor expressed on myeloid cells 2 (TREM2) is primarily expressed by myeloid cells, such as macrophages and microglia, and is related to phagocytosis, cytokine and complement production, and neuronal damage (12–15). Soluble TREM2 (sTREM2), which consists of the ectodomain of TREM2, can be detected in the serum and cerebrospinal fluid (CSF) (14). After TREM2 is shed from activated microglia and macrophages, its pro-inflammatory function is enhanced, and thus, an increase in the sTREM2 level represents strengthening of the inflammatory reaction (13, 14, 16). Elevated levels of sTREM2 have been observed in multiple neurologic diseases such as Alzheimer’s disease (AD), multiple sclerosis (MS), HIV infection, and neurosyphilis (17). Therefore, sTREM2 has been proposed as a biomarker for neuroinflammatory and neurodegenerative diseases (18). In addition, Goerres et al. observed the accumulation of microglia around the blood vessels in brain biopsy samples from PACNS patients (19). Therefore, we questioned whether microglia activation might contribute to the pathogenesis of PACNS as a neuroinflammatory disease.

The purpose of our study was to explore the correlation between sTREM2 levels and a clinical inflammatory index in PACNS patients to assess the diagnostic value of sTREM2 measurement and to investigate the pathogenesis of PACNS.

## Materials and methods

### Patients and design

Thirty-two patients treated in Beijing Tiantan Hospital between 2007 and 2021 were included in this cross-sectional study. The PACNS group consisted of 18 patients who were diagnosed with PACNS at acute stage according to the Birnbaum and Hellmann criterion. None of the 18 PACNS patients had symptoms of myelopathy, and 12 of them underwent lumbar puncture. The control group consisted of 14 patients who were diagnosed with non-inflammatory neurological diseases (3 with cerebral venous sinus thrombosis, 3 with benign intracranial hypertension, 2 with peripheral neuropathy, 2 with meninges carcinoma, 1 with diabetic retinopathy, 1 with intracranial hypotension headache, 1 with Paget disease of bone, and 1 with anxiety disorder). This study was approved by the Ethics Committee of Beijing Tiantan Hospital affiliated with Capital Medical University, and informed consent was obtained from all participants. The modified Rankin scale (mRS), National Institutes of Health Stroke Scale (NIHSS), and activities of daily living (ADL) scores were determined for evaluation of the severity of disease. Cerebral lesion volume was computed from MRI sequences collected during the active stage of PACNS.

We classified outcome at the time of hospital discharge as poor prognosis (mRS score ≥3 or relapse) and good prognosis (mRS score <3 and no relapse) after receiving treatments for 6 months. Relapse was defined as reoccurrence or worsening of neurological syndrome, or enlargement of the lesion area on MRI (10).

### Measurement of serum and CSF concentrations of sTREM2 and related cytokines

Serum (n=18) and CSF (n=12) samples from PACNS patients as well as serum (n=14) and CSF (n=14) samples from control patients were obtained during the acute stage. Supernatant from each sample was collected following centrifuged and stored at –80℃ until analysis. The sTREM2 concentration in each sample was measured using an enzyme-linked immunosorbent assay (ELISA) kit (abcam, Shanghai). The concentrations of tumor necrosis factor-α (TNF-α), interleukin-6 (IL-6), IL-8, IL-1β and complement component 4 (C4) were also measured in serum (n=13) and CSF (n=9) samples from PACNS patients using ELISA kits (abcam, Shanghai). All kits were employed following the manufacturer’s instructions.

### Routine CSF and serum measurement

The amount of White Blood Cells (WBC) were counted by Fuchs Rosenthal counting chamber. We tested the levels of CSF total protein, and serum and CSF immunoglobulin G (IgG) and albumin by using immunonephelometry. QAlb was calculated by using the formula: QAlb=CSF Albumin/Serum Albumin, which was used to assess the permeability of blood-brain barrier (BBB). We calculated a maximum normal QAlb (QNorm) according to the age of patients (QNorm=[(age/15)+4]x 10^-3^. We classified the PACNS patients as patients with increased QALb group according to QALb>QNorm.

### MRI analysis

All PACNS patients underwent MRI scanning of the brain before treatment with the same 3T Signa scanner (Philips Ingenia CX, The Netherlands). The details of the MRI sequences were: 1) T1-weighted (repetition time [TR]=6.45 ms, time to echo [TE]=2.98 ms, slice thickness=6.0 mm, matrix=512×512), 2) T2-weighted (TR=4600 ms, TE=106.28 ms, slice thickness= 6.0 mm, matrix=512×512), 3) fluid attenuation inversion recovery (FLAIR; TR=4800 ms, TE=57.95 ms, slice thickness= 6.0 mm, matrix=512×512), and 4) post-contrast T1-weighted (TR=6.45 ms, TE=2.98 mm, slice thickness=6 mm, matrix=512×512). The cerebral lesion of PACNS was defined as the region of hyperintensity on T2-weighted and FLAIR images with enhancement, and each lesion was identified by two neuroradiologists (Cong Hengri and Wang Xiaochen) who were blinded to the experimental and clinical data of patients. The cerebral lesion volume was calculated on MR images by Slicer software (Version 4.11).

### Statistical analysis

All statistical analyses were conducted using SPSS (Version 20) and Prism (Graphpad Version 9.0) software. The significance of differences between the different groups was analyzed using independent-samples t test and Mann-Whitney U test. Correlations between sTREM2 level and the levels of cytokines (except IL-6 and IL-1β in serum samples) and C4; the mRS, NIHSS, and ADL scores; as well as WBC, CSF total protein, QAlb, age and cerebral lesion volume were analyzed using Pearson’s two-tailed correlation or partial correlation analyses after controlling for age. Correlations between sTREM2 level and the levels of IL-6 and IL-1β in blood were analyzed using Spearman’s two-tailed correlation. A value of *P*<0.05 was defined as significant.

## Results

### Clinical characteristics and outcomes

The clinical, laboratory and imaging data for patients in the PACNS group (n=18) and those in the control group (n=14) with non-inflammatory neurological disease are presented in **Tables 1** and **2**. The clinical and laboratorial data between PACNS patients with and without increased QAlb was shown in **Table 3**. The most common clinical manifestations of PACNS were epileptic seizure (66.7%), focal neurological deficits (61.1%), consciousness dysfunction (55.6%), cognitive dysfunction (44.4%), headache (44.4%), and psychiatric symptoms (22.2%). In 100% of the PACNS patients were vascular-related hyperintense FLAIR and T2 lesion with gadolinium enhancement whose performance of MRI image conformed to imaging criteria of PACNS. There were 12 patients undergoing brain biopsy, and their pathological results supported the criteria of disease. The outcome of PACNS patients was defined according to the mRS score and the occurrence of relapse after at least 6 months of follow up. A poor outcome was defined by an mRS score >2 after treatment with immunotherapy for 6 months or by relapse. According to the follow-up data for the PACNS patients, eight patients (44.4%, 8/18) experienced a poor outcome.

**Table 1:** Clinical and laboratorial data of patients in the PACNS and control groups.

Subject details	PACNS (n=18)	Control (n=14)
Gender^a^ NO.(%)		
Male	14(77.8%)	7(50%)^ns^
Female	4(22.2%)	7(50%)^ns^
Age^b^(years, mean ± SD)	(33.00 ± 9.08)	(37.14 ± 9.57)^ns^
Clinic Symptoms n(%)		
Headache	8(44.4%)	–
Focal neurological deficits	10(55.6%)	–
Cognitive dysfunction	9(50%)	–
epileptic seizure	12(66.7%)	–
consciousness dysfunction	10(55.6%)	–
psychiatric symptoms	4(22.2%)	–
mRS score(mean ± SD)	(1.44 ± 1.69)	–
NIHSS(mean ± SD)	(3.00 ± 3.41)	–
ADL(mean ± SD)	(85.28 ± 22.13)	–
Follow-up time(month, mean ± SD)	(7.28 ± 1.07)	–
Good prognosis n(%)	10(55.6%)	–
Bad prognosis n(%)	8(44.4%)	–
CSF paramaters(n=12)		
WBC count>5 cells/mL, Total protein concentration>45mg/dL **n(%)**	4(33.3%)	–
IgG index(mean ± SD)	(0.47 ± 0.13)	–
QAlb(mean ± SD)	(5.33 ± 2.26)	–
CNS-specific OCBs **n(%)**	2(16.7%)	–
Brain biopsy n(%)	12(66.7%)	–

a Chi-squared test; ^b^Independent-samples t test; ^ns^No significance.

Data are expressed as mean ± SD as appropriate. PACNS, primary angiitis of the central nervous system; mRS, modified Rankin scale; NIHSS, National Institutes of Health Stroke Scale; ADL, Activities of Daily Living; CSF, cerebrospinal fluid; WBC, white blood cell; IgG, Immunoglobulin G; QAlb=CSF Albumin/Serum Albumin x 10^3^; CNS, central nervous system; OCBs, Oligoclonal bands.

**Table 2:** Imaging data and sTREM2 and inflammatory factors levels of patients in the PACNS and control groups.

Subject details	PACNS (n=18)	Control (n=14)
Brain magnetic resonance imaging		
Abnormal n(%)	18(100%)	–
Multiple hyperintense FLAIR lesions n(%)	18(100%)	–
Multiple bilateral infarctions n(%)	4(22.2%)	–
Intracranial hemorrhage n(%)	10(55.6%)	–
Cerebral lesion volume(mm^3, mean ± SD)	(92854.61 ± 100651.17)	–
Abnormal lesion enhancement on MRI n(%)	18(100%)	–
Serum sTREM2^a^(ng/mL, mean ± SD)	(26.81 ± 16.14)	(12.46 ± 3.76)*
CSF sTREM2^b^(ng/mL, mean ± SD)	(29.13 ± 17.01)	(14.26 ± 7.89)*
CSF cytokines(n=9)		
CSF TNFα(pg/mL, mean ± SD)	(6.36 ± 2.78)	–
CSF IL-6(pg/mL, mean ± SD)	(2.85 ± 0.99)	–
CSF IL-8(pg/mL, mean ± SD)	(65.73 ± 50.18)	–
CSF IL-1β(pg/mL, mean ± SD)	(7.22 ± 1.47)	–
Serum cytokines and complement(n=13)		
Serum TNFα(pg/mL, mean ± SD)	(15.28 ± 14.26)	–
Serum IL-6(pg/mL, mean ± SD)	(2.20 ± 0.43)	–
Serum IL-8(pg/mL, mean ± SD)	(73.37 ± 75.68)	–
Serum IL-1β(pg/mL, mean ± SD)	(6.17 ± 2.90)	–
Serum C4(g/L, mean ± SD)	(0.23 ± 0.07)	–

a Mann-Whitney U test; ^b^Independent-samples t test; *p<0.05.

Data are expressed as mean ± SD as appropriate. PACNS, primary angiitis of the central nervous system; FLAIR, fluid attenuation inversion recovery; CSF, cerebrospinal fluid; sTREM2, soluble triggering receptor expressed on myeloid cells 2; TNF-α, tumor necrosis factor α; IL-6, interleukin-6; IL-8, interleukin-8; IL-1β, interleukin-1β; C4, complement component 4.

**Table 3:** Difference about clinical and Laboratorial data between PACNS patients with and without increased QAlb.

Subject details	With increased QAlb(n=5)	Without increased QAlb(n=7)	P value
Gender^a^ NO(%)			
Male	4(80%)	6(85.7%)	0.793
Female	1(20%)	1(14.3%)	
**Age^b^(years, mean ± SD)**	(33.00 ± 6,78)	(30.14 ± 8.36)	0.544
**mRS score^c^(mean ± SD)**	(3.20 ± 1.64)	(1.00 ± 1.41)	0.030
**NIHSS^c^(mean ± SD)**	(6.60 ± 3.36)	(1.57 ± 2.37)	0.030
**ADL^c^(mean ± SD)**	(66.00 ± 26.55)	(94.29 ± 9.32)	0.048
Prognosis^a^(n,%)			
Good	2(40%)	3(42.9%)	0.921
Bad	3(60%)	4(57.1%)	
CSF paramaters			
WBC^b^(cells/mL, mean ± SD)	(6.60 ± 1.67)	(3.00 ± 1.91)	0.007
Protein^c^(mg/dL, mean ± SD)	(36.42 ± 11,79)	(23.40 ± 9.37)	0.030
IgG index^b^	(0.47 ± 0.12)	(0.47 ± 0.15)	0.958
QAlb^b^	(7.57 ± 1.08)	(3.73 ± 1.19)	<0.001
Cerebral lesion volume^b^(mm^3, mean ± SD)	(193501.40 ± 132546.77)	(69586.71 ± 61791.10)	0.106
**Serum sTREM2^b^(ng/mL,mean ± SD)**	(42.65 ± 22.05)	(19.05 ± 7.51)	0.073
**CSF sTREM2^b^(ng/mL, mean ± SD)**	(32.06 ± 17.78)	(27.03 ± 17.52)	0.636
CSF cytokines			
CSF TNFα^b^(pg/mL, mean ± SD)	(6.70 ± 2.66)	(6.09 ± 3.14)	0.726
CSF IL-6^b^(pg/mL, mean ± SD)	(3.22 ± 0.89)	(2.76 ± 1.43)	0.590
CSF IL-8^b^(pg/mL, mean ± SD)	(74.33 ± 56.95)	(58.86 ± 49.70)	0.676
CSF IL-1β^b^(pg/mL, mean ± SD)	(6.42 ± 0.47)	(7.86 ± 1.74)	0.155
Serum cytokines and complement			
Serum TNFα^b^(pg/mL, mean ± SD)	(23.75 ± 21.69)	(15.13 ± 10.81)	0.506
Serum IL-6^c^(pg/mL, mean ± SD)	(2.35 ± 0.7)	(2.00 ± 0)	0.556
Serum IL-8^c^(pg/mL, mean ± SD)	(106.74 ± 78.91)	(68.90 ± 97.37)	0.730
Serum IL-1β^c^(pg/mL, mean ± SD)	(8.80 ± 4.50)	(5.00 ± 0)	0.286
Serum C4^b^(g/L, mean ± SD)	(0.249 ± 0.80)	(0.213 ± 0.066)	0.480

a Chi-squared test

b Independent-samples t test

c Mann-Whitney U test.

Data are expressed as mean ± SD as appropriate. mRS, modified Rankin scale; NIHSS, National Institutes of Health Stroke Scale; ADL, Activities of Daily Living; CSF, cerebrospinal fluid; WBC, white blood cell; IgG, Immunoglobulin G; QAlb=CSF Albumin/Serum Albumin x 10^3^; sTREM2, soluble triggering receptor expressed on myeloid cells 2; TNF-α, tumor necrosis factor α; IL-6, interleukin-6; IL-8, interleukin-8; IL-1β, interleukin-1β; C4, complement component 4.

### Comparison of sTREM2 levels between PACNS and control patients

**Figure 1:**
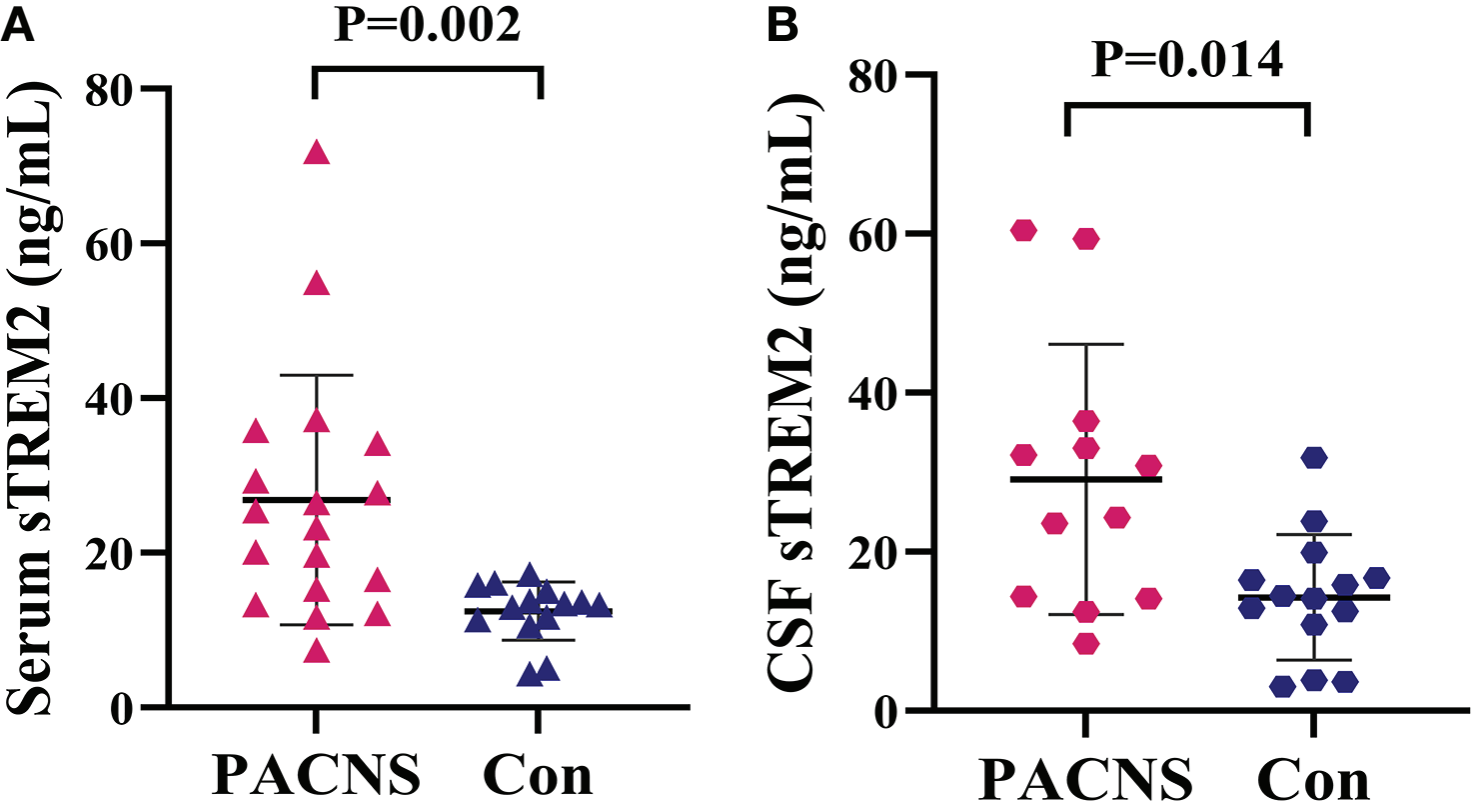
Elevated sTREM2 concentrations in the serum and CSF of PACNS patients. **(A, B)** sTREM2 concentrations in serum samples (n=18) and CSF samples (n=12) from the PACNS group were significantly higher than those in samples from the control group (n=14).

The concentration of sTREM2 in serum samples from the PACNS group was significantly higher than that in serum samples from the control group (*P=*0.002, **Figure 1A**). The concentration of sTREM2 in CSF samples from the PACNS group also were significantly higher than that in CSF samples from the control group (*P=*0.014, **Figure 1B**).

### Correlations between sTREM2 levels and measures of PACNS severity

**Figure 2:**
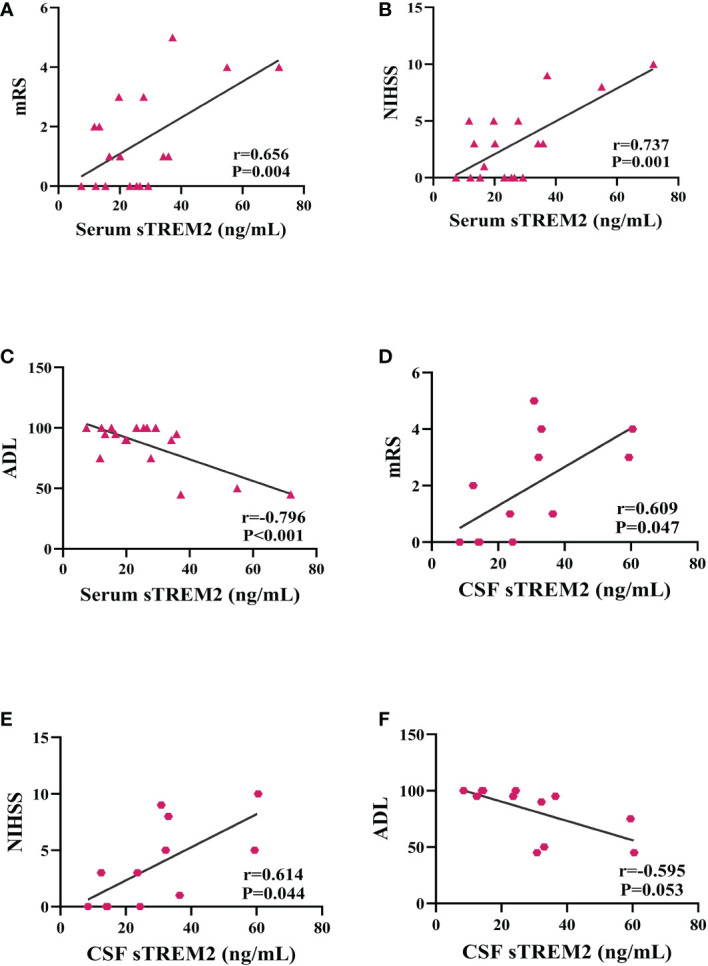
Serum and CSF sTREM2 levels were correlated with mRS, NIHSS, and ADL scores as well as outcome at 6-month follow-up in the PACNS group. Partial correlation analyses after controlling for age. **(A–C)** In serum samples (n=18), positive correlations were found between the serum sTREM2 concentration and the mRS score and NIHSS score, and a negative correlation was found between the serum sTREM2 concentration and the ADL score. **(D–F)** In CSF samples (n=12), mildly positive correlations were found between the CSF sTREM2 concentration and the mRS and NIHSS scores, and a negative correlation was observed between the CSF sTREM2 concentration and the ADL score.

In PACNS patients, positive correlations were observed between the serum sTREM2 level and the mRS score (r=0.656, *P*=0.004, **Figure 2A**) and NIHSS score (r=0.737, *P*=0.001, **Figure 2B**), and a negative correlation was observed between the serum sTREM2 level and the ADL score (r=-0.796, *P<*0.001, **Figure 2C**). Relatively milder positive correlations were found between the CSF sTREM2 level and mRS score (r=0.609, *P*=0.047, **Figure 2D**) and NIHSS score (r=0.614, *P*=0.044, **Figure 2E**) in PACNS patients, and the CSF sTREM2 level was a possible trend toward significance with the ADL score (r=-0.595, *P*=0.053, **Figure 2F**).

### Correlations between sTREM2 levels and inflammatory factors levels in PACNS patients

**Figure 3:**
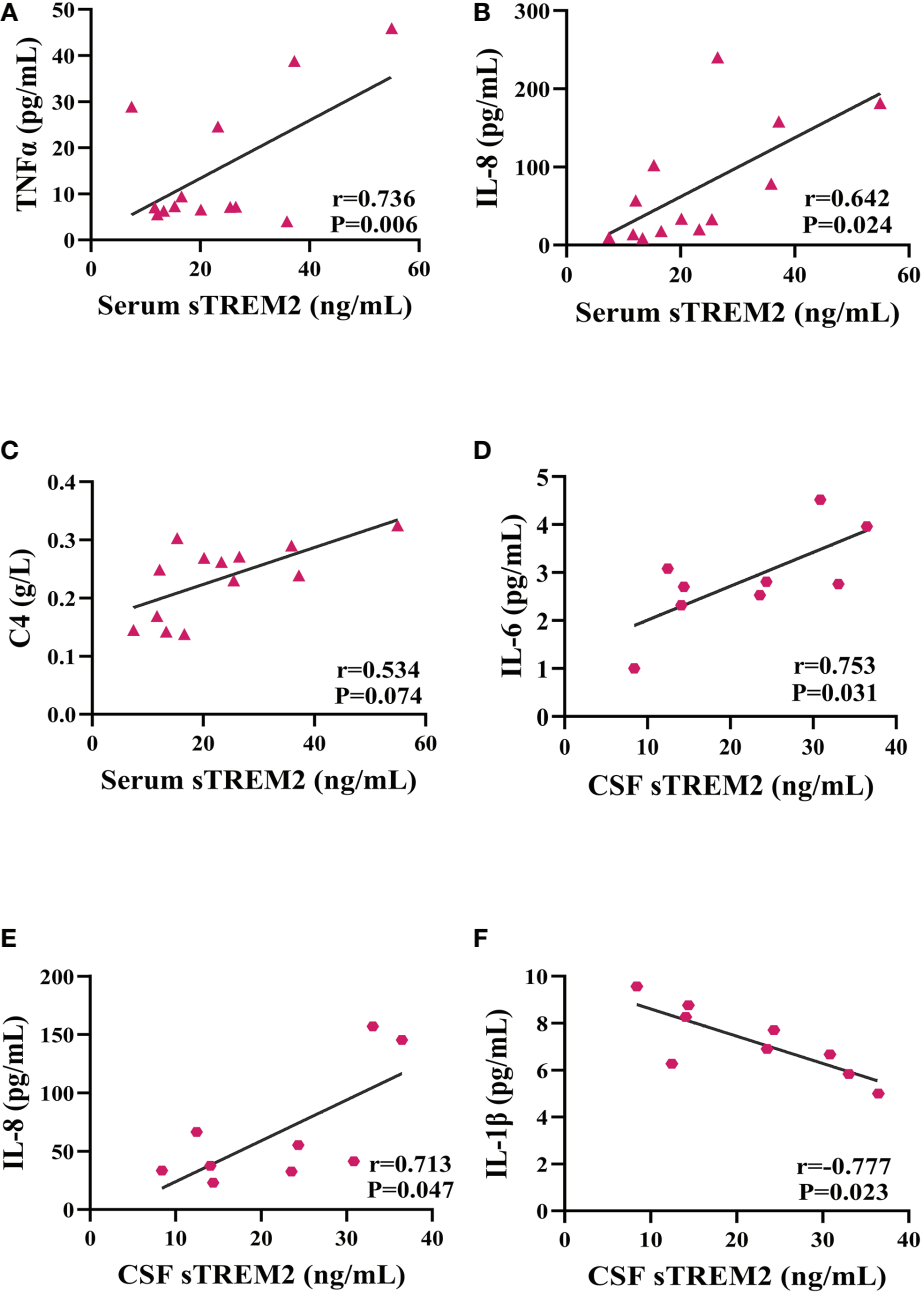
sTREM2 levels correlated with neuroinflammatory factor levels. Partial correlation analyses after controlling for age. **(A–C)** In the serum samples of PACNS patients (n=13), the sTREM2 concentration was positively correlated with the TNFα, IL-8 and C4 concentrations. **(D–F)** In the CSF samples of PACNS patients (n=9), the sTREM2 concentration was positively correlated with the IL-6 and IL-8 concentrations and negatively correlated with the IL-1β concentration.

The serum sTREM2 concentration of PACNS patients was positively related with the serum TNF-α concentration (r=0.736, *P*=0.006, **Figure 3A**) as well as the serum IL-8 concentration (r=0.642, *P*=0.024, **Figure 3B**). The data revealed a nearly significant correlation between the serum sTREM2 and C4 levels (r=0.534, *P*=0.074, **Figure 3C**) and no correlations between the serum sTREM2 concentration and serum IL-6 and IL-1β concentrations (r=0.182, *P*=0.552; r=0.079, *P*=0.798). In addition, the CSF sTREM2 level of the PACNS patients was significantly positively correlated with the CSF IL-6 concentration (r=0.753, *P*=0.031, **Figure 3D**) and CSF IL-8 concentration (r=0.713, *P*=0.047, **Figure 3E**), and negatively correlated with the CSF IL-1β concentration (r=-0.777, *P*=0.023, **Figure 3F**). No correlation was observed between the CSF levels of sTREM2 and TNF-α (r=0.109, *P*=0.78).

### Association of sTREM2 levels and cerebral lesion size in PACNS patients

**Figure 4:**
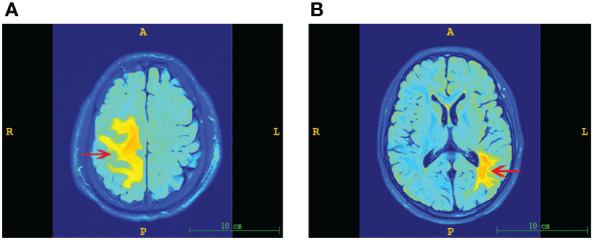
Representative MR images of cerebral lesions of varying size in PACNS patients. Dominant lesions are marked by arrows. **(A)** Image of a larger lesion showing hyperintense regions on FLAIR imaging. **(B)** Image of a smaller lesion observed on FLAIR imaging.

**Figure 5:**
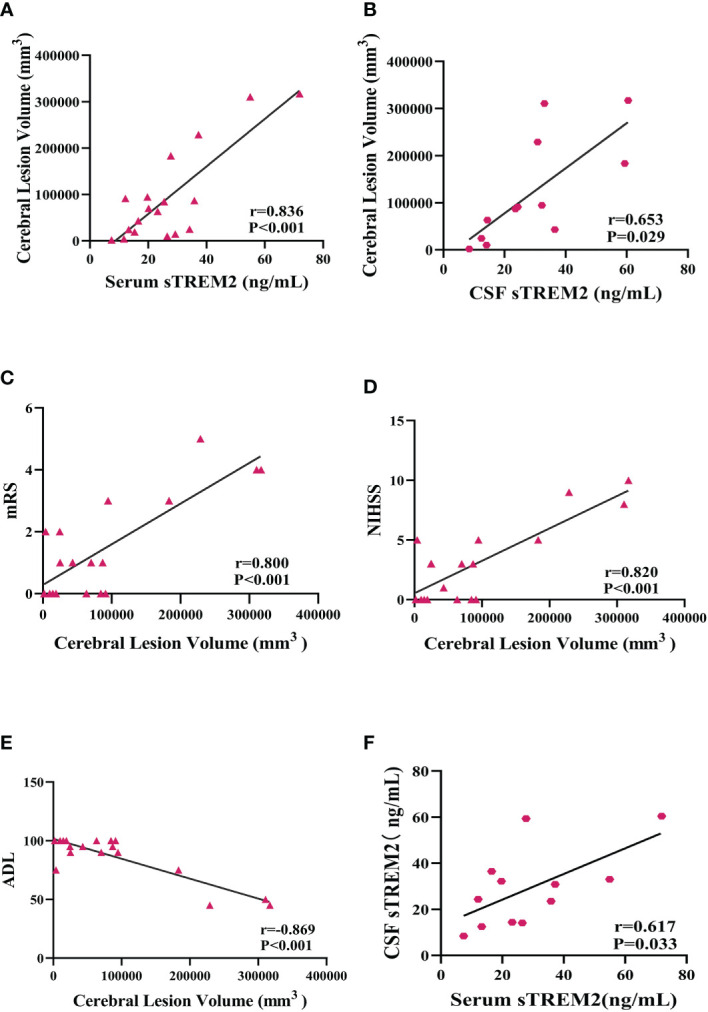
Serum and CSF sTREM2 levels were correlated with cerebral lesion volume in PACNS patients, and cerebral lesion volume was associated with mRS, NIHSS, and ADL scores. **(A, B)** Serum (n=18) and CSF (n=12) sTREM2 concentrations were significantly correlated with cerebral lesion volume analysed by partial correlation analyses after controlling for age. **(C–E)** The cerebral lesion volume in PACNS patients (n=18) was strongly correlated with the mRS, NIHSS, and ADL scores. **(F)** The serum (n=12) sTREM2 levels are positively correlated with CSF (n=12) sTREM2 levels.

The cerebral lesion size in PACNS patients varied on MRI (**Figure 4**). From the results of MR image analysis for PACNS patients, the serum sTREM2 level was found to be significantly positively correlated with cerebral lesion volume (r=0.836 *P*<0.001, **Figure 5A**). Additionally, the CSF sTREM2 level was significantly associated with cerebral lesion volume (r=0.653, *P*=0.029, **Figure 5B**). Moreover, cerebral lesion volume in PACNS patients was found to be strongly correlated with the mRS score (r=0.800, *P*<0.001, **Figure 5C**), NIHSS score (r=0.820, *P*<0.001, **Figure 5D**), and ADL score (r=-0.869, *P*<0.001, **Figure 5E**). Additionally, the results showed serum sTREM2 levels are positively correlated with CSF sTREM2 levels(r=0.617, P=0.033, **Figure 5F**).

### Correlation between sTREM2 levels and prognosis of PACNS patients

**Figure 6:**
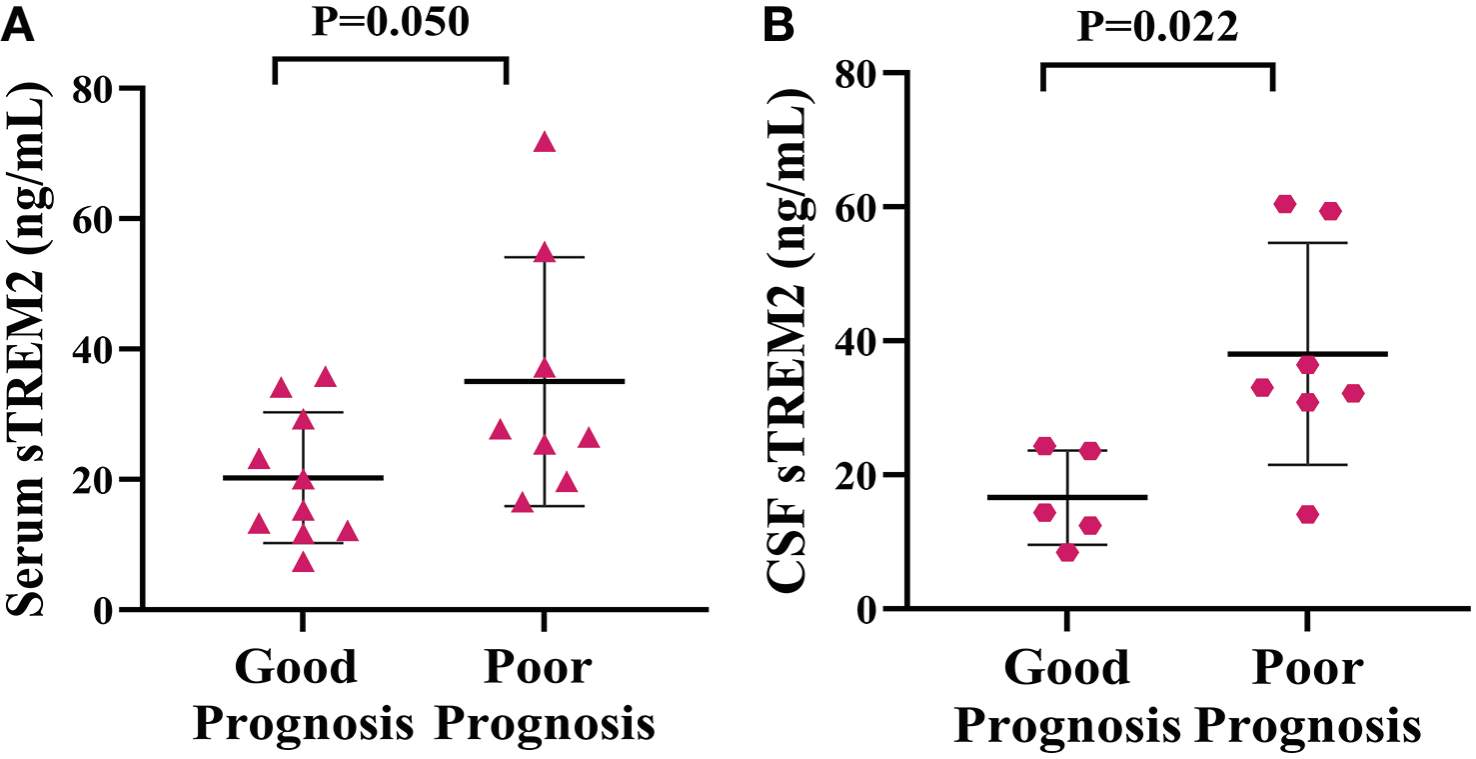
Elevated serum and CSF sTREM2 concentrations were linked to a poor prognosis in PACNS patients. **(A)** The serum sTREM2 concentration in PACNS patients (n=18) was significantly higher in patients with a poor prognosis (n=6) than in those with a good prognosis (n=12). **(B)** The CSF sTREM2 concentration in PACNS patients (n=12) also appeared to be significantly higher in patients with a poor prognosis.

Analysis of the 6-month follow-up data for PACNS patients revealed that the serum sTREM2 concentration was on the boundary of significant higher in PACNS patients with a poor prognosis than in those with a good prognosis (*P*=0.050, **Figure 6A**). Consistently, the CSF sTREM2 concentration appeared to be significantly higher in PACNS patients with poor prognosis (*P*=0.022, **Figure 6B**).

### Association of sTREM2 levels with cognitive function and age in PACNS patients

**Figure 7:**
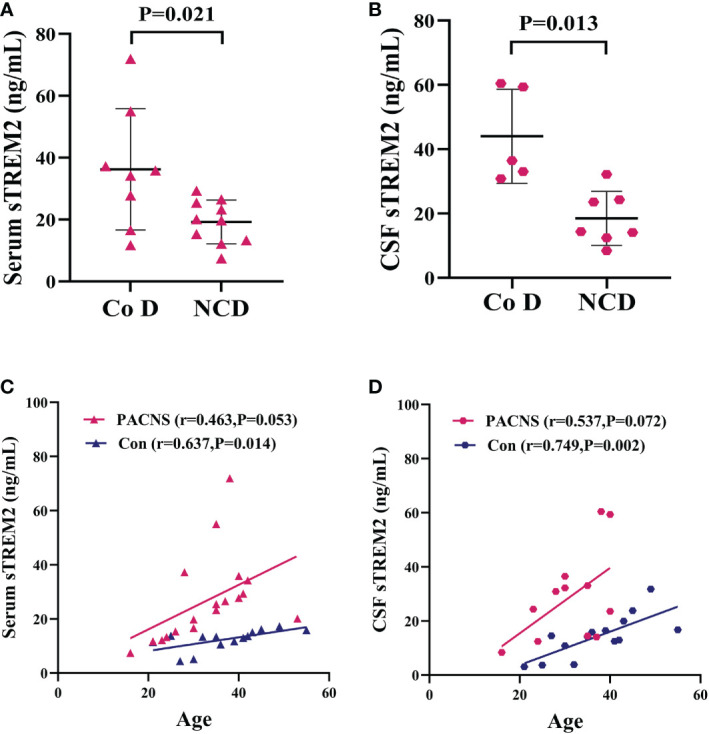
Elevated serum and CSF sTREM2 concentrations in PACNS patients were associated with cognitive dysfunction and patient age. Co D, cognitive dysfunction. NCD, non cognitive dysfunction. **(A, B)** In the serum (n=18) and CSF (n=12) samples, sTREM2 concentrations were higher in PACNS patients with cognitive dysfunction that in those without. **(C)** In the serum samples, the sTREM2 concentration in PACNS (n=18) was correlated with patient age, whereas the sTREM2 concentrations in control (n=14) were significantly related to age. **(D)** In CSF samples, this correlation in PACNS group (n=13) approached significance, and the CSF sTREM2 concentration in control group (n=14) showed a strong correlation with age.

The serum and CSF sTREM2 concentration were higher in PACNS patients with cognitive dysfunction than in those without (*P*=0.021, *P*=0.013; **Figures 7A, B**). The serum sTREM2 concentration showed a mild correlation with PACNS patient age (r=0.463, *P*=0.053, **Figure 7C**), and the serum sTREM2 concentration was significantly associated with control patients age (r=0.637, *P*=0.014, **Figure 7C**). In addition, this correlation for the CSF sTREM2 concentration in PACNS group approached significance (r=0.537, *P*=0.072, **Figure 7D**), and the CSF sTREM2 concentration showed a strong correlation with control patient age (r=0.749, *P*=0.002, **Figure 7D**).

### Association of QAlb and biomarker of CNS inflammation in PACNS

**Figure 8:**
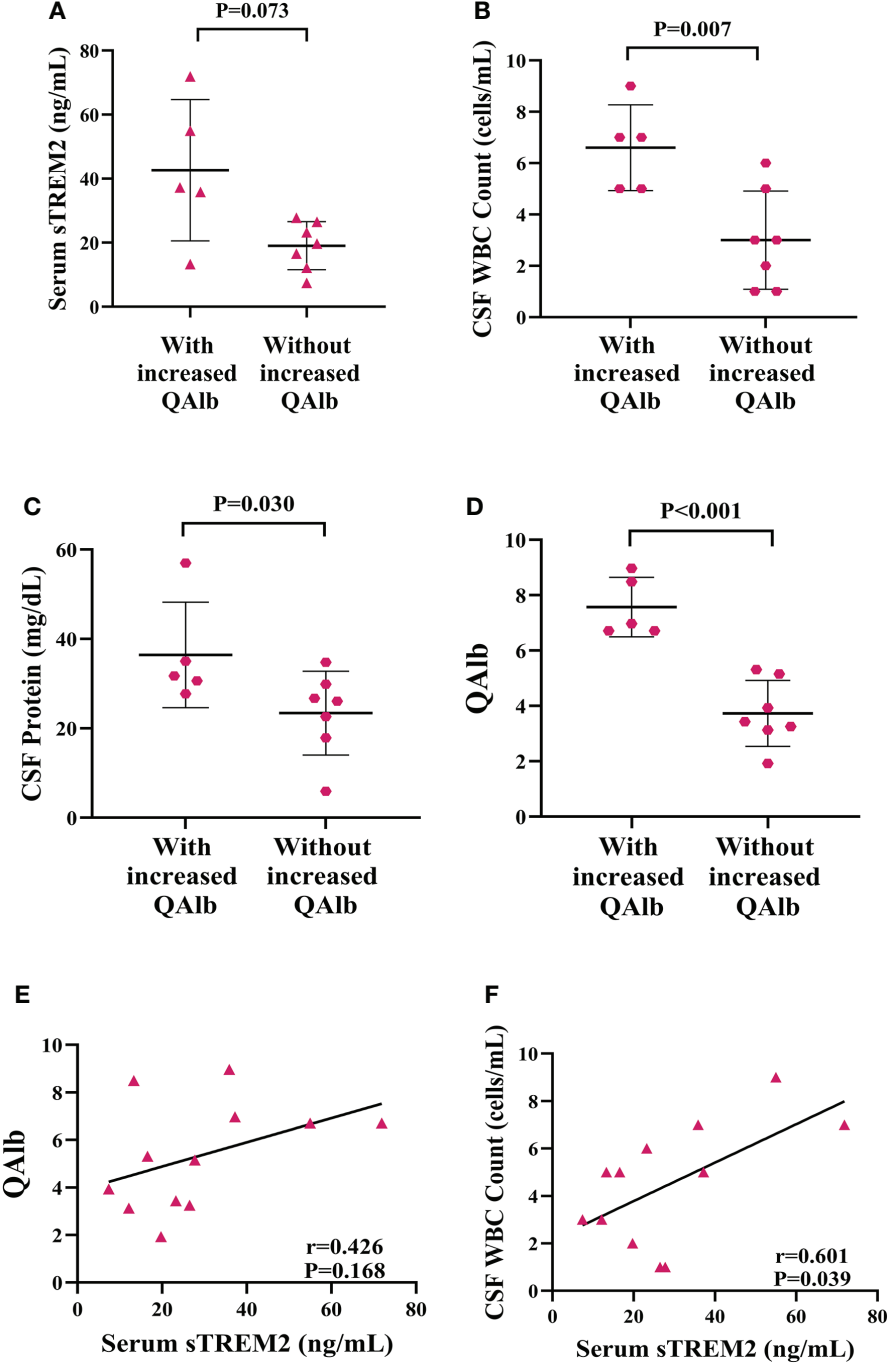
Elevated levels of biomarker of CNS inflammation in PACNS patients with increased QAlb. **(A–D)** In the PACNS patients with increased QAlb (n=5), levels of CSF WBC count, CSF total protein, and QAlb appeared to be significantly higher than in those without (n=7). **(E, F)** In the serum samples (n=12), there was a positive correlation between serum sTREM2 and WBC amount in CSF and a very slight trend toward significance with QAlb.

The concentration of sTREM2 in serum samples from patients with increased QAlb group was nearly significantly higher than patients without increased QAlb (*P=*0.073, **Figure 8A**). The amount of WBC in CSF samples from patients with increased QAlb was significantly more than patients without increased QALb group (*P=*0.007, **Figure 8B**). The CSF total protein concentration appeared to be significantly higher in PACNS patients with increased QALb (*P=*0.030, **Figure 8C**). The amounts of QAlb were higher in PACNS patients with increased QAlb than in those without (*P<*0.001, **Figure 8D**). There was a very slight trend toward significance between serum sTREM2 and QAlb (CSF albumin/serum albumin ratio) (r=0.426, P=0.168, **Figure 8E**). There was a positive correlation between serum sTREM2 and WBC amount in CSF (r=0.601, P=0.039, **Figure 8F**). No correlation was observed between the serum levels of sTREM2 and CSF protein (r=0.152, *P*=0.637). CSF sTREM2 levels were not significantly related to amount of WBC in CSF, levels of CSF protein and QAlb (r=0.071, P=0.827; r=0.137, P=0.668; r=0.145, P=0.654).

### Correlation between QAlb and measures of PACNS severity

**Figure 9:**
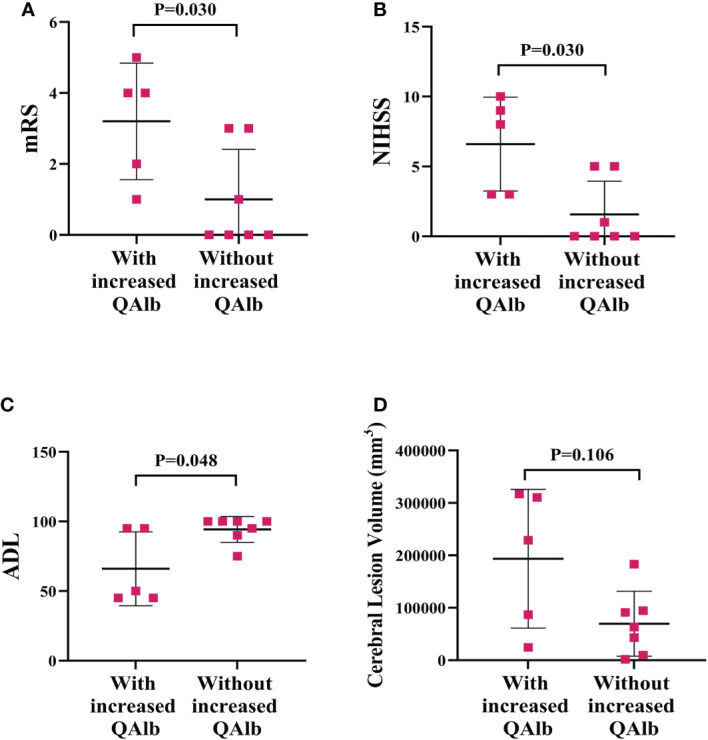
More serious disease performance and cerebral lesion volume in PACNS patients with increased QAlb. **(A–C)** The mRS and NIHSS were significantly higher and ADL was significantly lower in PACNS patients with increased QAlb (n=5) than patients without increased QAlb (n=7). **(D)** In the PACNS patients with increased QAlb (n=5), levels of cerebral lesion volume appeared to be significantly higher than in those without (n=7).

The mRS and NIHSS were significant higher and ADL was significant lower in PACNS patients with increased QAlb than in those without (*P*=0.030, *P*=0.030, *P*=0.048; **Figures 9A–C**). The cerebral lesion volumes were higher but not significant in PACNS patients with increased QAlb (*P=*0.106, **Figure 9D**).

## Discussion

The present study revealed elevated levels of sTREM2 in the serum and CSF of PACNS group compared with controls with non-inflammatory neurological diseases. Moreover, the analyses showed that sTREM2, as a biomarker, was significantly correlated with disease severity, imaging features and prognosis among PACNS patients. To our knowledge, this is the first report regarding altered sTREM2 levels in PACNS patients and the potential correlations with microglia/macrophage activation in the pathogenesis of PACNS, a contributor to disease severity and prognosis.

Microglia are important immunosurveillance cells of the CNS. Activated microglia participate in neuron inflammation and neuronal damage that causes clinical symptoms, and this may be a pathogenic mechanism of neuron-inflammatory disease (20). TREM2 as a transmembrane protein receptor is expressed by microglia and macrophages, and combined with DAP12 complex, TREM2 inhibits the maturation of immune cells and production of inflammatory factors (21). In mouse microglia and macrophages, knockdown of TREM2–DAP12 enhances the transcription of genes related to production of pro-inflammatory cytokines, such as TNF-α, IL-6 and IL-1β (13, 14, 16). Therefore, activated microglia and macrophages shed TREM2 to down-regulate DAP12 expression and promote the inflammatory reaction, and the soluble variant TREM2 (sTREM2) can be detected in the CSF and serum, which is the foundation for the experimental design of the present study (22). Based on biopsy findings, PACNS is an inflammatory disease of the CNS, and microglia are found around CNS vessels. Moreover, positron emission tomography (PET) using PK11195 (1-[2-chlorophenyl]-N-methyl-N-[1-methylpropyl] -3-isoquinoline carboxamide) as an indicator of microglial activation also showed a potential role for microglia in the pathogenic mechanism of PACNS (2, 19). Therefore, we hypothesized that microglia contribute to the pathogenesis of PACNS, and our finding that the sTREM2 concentration is elevated in the CSF of PACNS patients supported the involvement of microglia in the pathogenesis of PACNS.

Our results show the correlation of sTREM2 concentration in periphery and sTREM2 levels in CNS. It reveals the might role of activated microglia and macrophage participating in the inflammation reaction both in the CNS and periphery in PACNS. The roles of activated microglia and macrophages in promoting inflammation *via* increased production of cytokines and complement components are well established (13, 20, 23). The results of the present study also showed the sTREM2 levels in PACNS patients were positively correlated with the levels of pro-inflammatory cytokines (TNFα, IL-6, IL-8, IL-1β) and complement (C4). Activation of microglia enhances the generation of cytokines, chemokines, and matrix metalloproteinases (MMPs), which destroy the blood–brain barrier (BBB), allowing blood-derived cytokines and complement components as well as other immune cells to enter the CNS, which leads to further activation of microglia and a continued cascade of cytokine and complement increases that promotes damage to neurons. Meanwhile, activated macrophages in the peripheral blood secrete high levels of blood-derived inflammatory factors that pass through the damaged BBB into the CNS to promote CNS inflammation (24). Our imaging and clinical data reveal a mechanism by which microglia and macrophage activation represented by elevated sTREM2 levels causes a more severe inflammatory reaction in vessels by promoting production of pro-inflammatory factors production. These inflammatory factors attack the endothelium, which aggravates the necrosis of cells within the vessels wall, causing the expansion of the area of ischemia within brain tissues, thereby enlarging the cerebral lesion. Meanwhile, a larger lesion corresponds to more neurons being damaged and cuases more serious clinical syndrome governed by impaired neurons. In summary, the higher sTREM2 levels cause increased production of inflammatory factors, which leads to the formation of a larger lesion in the CNS and more severe clinical symptoms in PACNS patients. In addition, higher sTREM2 levels were observed in PACNS patients with poor outcomes, which is considered to be because of the weakening of the microglia functions of tissue repair, phagocytosis of dying cells, and control local inflammation, after shedding of TREM2, leading to damaging the functional impairment of the repaired nerves repair (25). However, our results also showed that sTREM2 levels were not related to some cytokines and were negatively correlation with levels of the pro-inflammatory cytokines IL-1β, likely due to the small sample size in our study because of limited amounts of samples. Therefore, additional research we need with larger sample sizes and further analyses are needed to confirm these correlations.

We analysed the data of the PACNS with increased QAlb to investigate the putative role of increased BBB in the inflammation reaction of PACNS. Our results showed that the levels of serum sTREM2, WBC, protein, disease severity score and cerebral lesion volume elevate when the patients have increased QAlb. QAlb is deemed to be an indicator of assessing the permeability of BBB, it can be influenced by many factors, such as the altered CSF flow and so on, though (26, 27). The above results indicated that increased BBB permeability is associated with increased serum sTREM2 levels, and more inflammatory factors entered the CNS, which enlarged the cerebral lesion and aggravated the severity of disease. And our results showed a very slight trend toward significance between serum sTREM2 and QAlb, it also suggested that BBB breakdown may be the feature of PACNS. But there were no significance between CSF sTREM2 levels and QAlb levels. The results so far are not enough to prove this conclusion, and we need further studies on the pathogenesis of PACNS.

Our analysis of the clinical characteristics of PACNS patients revealed that sTREM2 levels were higher in PACNS patients who experienced cognitive dysfunction. Kleinberger et al. reported that loss of TREM2 leads to diminished clearance by microglia of amyloid β-peptide, which plays an important role in neurodegeneration and has been linked with the pathogenesis of AD (22, 28, 29). Therefore, the same mechanism may result in cognition impairment in PACNS. On the other hand, sTREM2 levels are known to increase with aging under both physiological and pathological conditions, in combination with the reduced ability of microglia to clear amyloid β-peptide and apoptotic neurons, indicating that sTREM2 is involved in the process of neurodegeneration (30, 31). The results of the present study also showed that sTREM2 levels correlated with patient age in both the PACNS and control groups, but the correlation was stronger in the control group. Together our findings demonstrate a role for TREM2 in the pathogenesis of PACNS based on abnormal increases in TREM2 in PACNS patients.

The present study has several limitations that must be considered. This was a single-center study, and thus, biases may exist due to the limited sample size and variation in follow-up period. Further studies on the pathogenesis of PACNS are needed to confirm and expand upon our findings.

## Conclusion

From the results of this study, we conclude that both serum and CSF sTREM2 levels are significantly elevated in PACNS patients. This indicates that sTREM2 is linked to the pathogenesis of PACNS. Moreover, sTREM2 levels were significantly positively associated with levels of pro-inflammatory factors, lesion volume, and indexes of disease severity in PACNS patients. Therefore, sTREM2 in CSF and serum may provide a valuable biomarker for monitoring disease severity, predicting lesion volume, and predicting prognosis in PACNS patients.

## Data availability statement

The original contributions presented in the study are included in the article/supplementary materials, further inquiries can be directed to the corresponding author/s.

## Ethics statement

The studies involving human participants were reviewed and approved by Ethics Committee of Beijing Tiantan Hospital. Written informed consent to participate in this study was provided by the participants’ legal guardian/next of kin. Written informed consent was obtained from the individual(s), and minor(s)’ legal guardian/next of kin, for the publication of any potentially identifiable images or data included in this article.

## Author contributions

All authors listed have made direct and indirect efforts. HW, HaC, TG and JM designed experiments, interpreted results, and drafted the manuscript of report. JS and YW participated in experimental operation and analysis of the data. YM and WX took part in clinical and image information collection. HeC was involved in MR images analyses. YK and QD were responsible for sample collection. XZ and LY provided administrative, technical, and material support. All authors contributed to the article and approved the submitted version.
